# Implications of Aging in Plastic Surgery

**DOI:** 10.1097/GOX.0000000000002085

**Published:** 2019-01-14

**Authors:** Danny S. Roh, Adriana C. Panayi, Shalender Bhasin, Dennis P. Orgill, Indranil Sinha

**Affiliations:** From the * Division of Plastic Surgery, Brigham and Women’s Hospital, Boston, Mass; † Division of Endocrinology, Brigham and Women’s Hospital, Boston, Mass.

## Abstract

Given the rapidly aging population, investigating the effect of age on plastic surgery outcomes is imperative. Despite this, the topic has received relatively little attention. Furthermore, there appears to be little integration between the basic scientists investigating the mechanisms of aging and the plastic surgeons providing the majority of “antiaging” therapies. This review first provides a description of the effects and mechanisms of aging in 5 types of tissue: skin, adipose tissue, muscles, bones and tendons, and nervous tissue followed by an overview of the basic mechanisms underlying aging, presenting the currently proposed cellular and molecular theories. Finally, the impact of aging, as well as frailty, on plastic surgery outcomes is explored by focusing on 5 different topics: general wound healing and repair of cutaneous tissue, reconstruction of soft tissue, healing of bones and tendons, healing of peripheral nerves, and microsurgical reconstruction. We find mixed reports on the effect of aging or frailty on outcomes in plastic surgery, which we hypothesize to be due to exclusion of aged and frail patients from surgery as well as due to outcomes that reported no postsurgical issues with aged patients. As plastic surgeons continue to interact more with the growing elderly population, a better appreciation of the underlying mechanisms and outcomes related to aging and a clear distinction between chronological age and frailty can promote better selection of patients, offering appropriate patients surgery to improve an aged appearance, and declining interventions in inappropriate patients.

## Abstract 2

Supplemental Digital Content is available in the text.

## INTRODUCTION

The United States Census Bureau estimates the population >65 years will be 88.5 million by 2050, a 105% increase from 2015.^1^ Despite this expanding demographic, the comprehensive effect of age on plastic surgical outcomes and postoperative rehabilitation has received little attention. As new research on aging mechanisms and novel therapies expand, there will need to be increased awareness within the plastic surgery community. As plastic surgeons encounter this growing aged population more, familiarity of basic mechanisms of aging combined with recognition of age impacts on surgical outcomes and rehabilitation becomes essential. This knowledge will help plastic surgeons to continue to obtain consistent results in a predictable manner and avoid morbidity and mortality in our growing elderly population.

## PHYSIOLOGIC AND TISSUE CHANGES WITH AGING

The aging process is variable and complex and multiple inherited factors, unique to individuals, contribute to aging. Individual organ systems and tissues differentially age and several anatomic factors lead to age-related changes in rather predictable manner. However, the individual experience with aging must always be considered in both preoperative and postoperative assessments (**see** video, Supplemental Digital Content 1, which displays the aging experience. Patient perspective on the impact of aging personal health and well-being. This video is available in the “Related Videos” section of the Full-Text article at PRSGlobalOpen.com or at http://links.lww.com/PRSGO/A957).

**Video Graphic 1.**
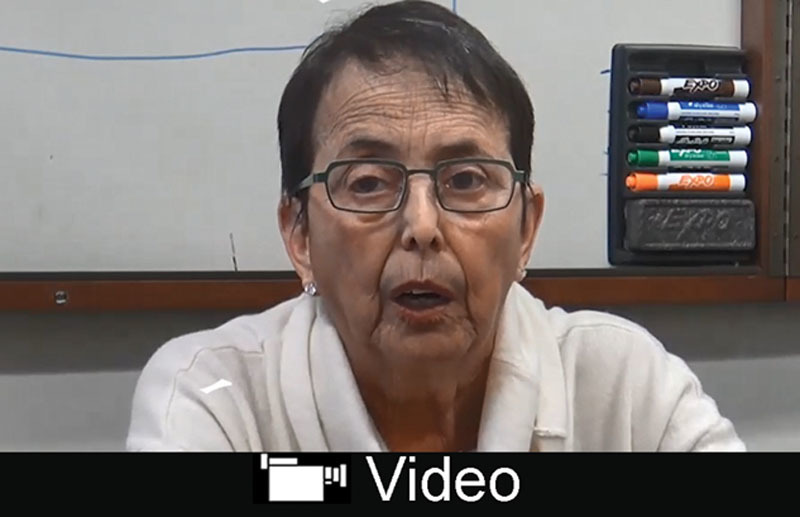
See video, Supplemental Digital Content 1, which displays the aging experience. Patient perspective on the impact of aging on personal health and well-being. This video is available in the “Related Videos” section of the Full-Text article at PRSGlobalOpen.com or at http://links.lww.com/PRSGO/A957.

### Skin Aging

**Fig. 1.**
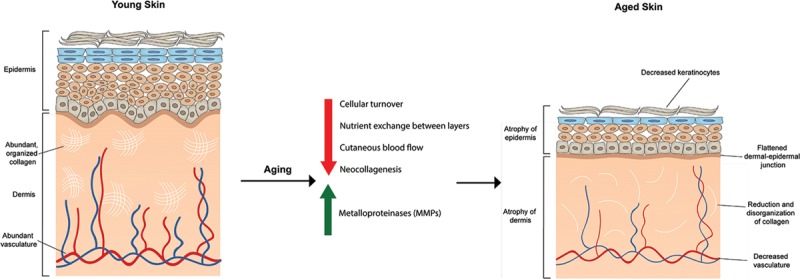
Skin aging. Decreased cellular turnover and inefficient nutrient exchange between the different layers occurring with aging result in atrophy of both the epidermis and dermis. The decrease in collagen number and organization result from decreased production of collagen as well as increased breakdown by metalloproteinases. A reduction in the vasculature is also seen leading to inefficient cutaneous blood supply.

Aged skin has epidermal thinning, decreased cellular turnover, and considerable atrophy.^2–5^ However, barrier function is largely unaltered.^3,6^ Keratinocyte proliferation declines, dermal-epidermal junctions flatten, nutrient exchange between layers is reduced, and there is increased fragility.^2–4^ Aged dermis undergoes thinning and atrophy, decrease in cellularity, vascularity, and extracellular matrix.^2,3,5,7^ Collagen fibrils become disorganized, fragmented, and reduced in number and diameter.^3–6^ Net collagen reduction results from increased metalloproteinases and decreased neocollagenesis by aged fibroblasts.^4,6^ Aged skin immune function is compromised with diminished Langerhans’s cells, decreased function of monocytes and macrophages, and overall immunological senescence^8^ (Fig. 1).

### Adipose Aging

**Fig. 2.**
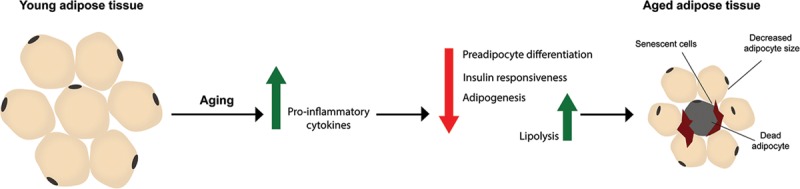
Adipose aging. The increased production of proinflammatory cytokines that occurs with aging inhibits preadipocyte differentiation leading to a decrease in adipocytes. Furthermore, there is a marked decrease in adipocyte size, an increase in senescent markers and cells and a shortening of the adipocyte telomeres. A decrease in insulin responsiveness promotes lipolysis and production of factors that inhibit adipogenesis.

Aged adipose tissue releases proinflammatory cytokines impairing preadipocyte differentiation necessary for regeneration.^9,10^ These cytokines cause also decrease adipocyte size, alter insulin responsiveness, and stimulate lipolysis.^9^ Senescence is increased in subcutaneous adipocyte.^11^ Aging redistributes fat toward visceral and ectopic deposition.^9,10^ This contributes to lipotoxicity and systemic dysfunction due to local effects within ectopic tissue.^4,9,10,12,13^ As adipose tissue is an increasing source of autologous transfers, understanding changes in aging fat has far-reaching clinical relevance (Fig. 2).

### Muscle Aging

**Fig. 3.**
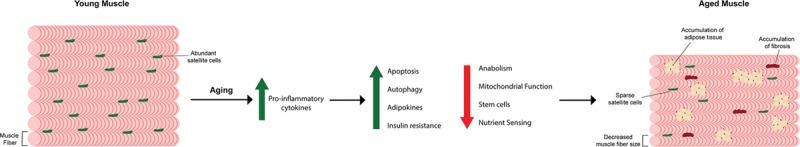
Muscle aging. Aging causes the release of proinflammatory cytokines that lead muscle to become atrophic through decreased anabolism, as well as increased autophagy and catabolism. Stem cell exhaustion results in a decrease in satellite cells, leading to a decline in the reparative capacity of damaged muscle and increased fibrosis. Proteostasis and a deregulation in nutrient sensing result in a decrease in fiber size, and mitochondrial dysfunction may lead to decreased endurance. An accumulation of adipose tissue within muscle promotes inflammation and insulin resistance.

Loss of muscle mass, declining strength, and physical function occurs with age.^14,15^ By 80 years, 30% of muscle mass is lost.^15^ Decreased muscle anabolism and increased expression of inflammatory factors contribute to skeletal muscle catabolism with increased apoptosis and decreased mitochondrial function.^14,15^ The reparative capacity of aged muscles is compromised by limited regenerative capacity of satellite cells, or muscle stem cells.^16^ Aged satellite cells diminish in number and function and eventually become senescent.^17^ These processes decrease muscle fiber cross-sectional area, delay repair, and accumulate fat and fibrosis within muscle leading to functional loss.^14–16^ Age-related motor unit loss results in atrophy and loss of myofibers^14,18^ (Fig. 3).

### Bone and Tendon Aging

Age-related changes in bone include loss of mass and mineral content, increased marrow fat content, reduced calcium and phosphate stores, and altered response to growth factors and hormones.^19^ Osteoblasts, osteoclasts, osteocytes, and progenitor cells become senescent with age.^13^ This contributes to osteoporosis and skeletal fragility, increasing susceptibility to fractures.^20^ The decline in structure and function of aged tendons results from degeneration of tenocytes and collagen fibers, accumulation of lipids and ground substance, and calcium deposits. Tenoblast metabolic activity decreases with age reflective of impaired healing. Overall, this results in tensile strength loss, stiffness, increased susceptibility to damage, and impaired healing.^21,22^

### Nerve Aging

Aged peripheral nerves undergo anatomic and physiological deterioration. Aged Schwann cells have diminished repair responses impairing regeneration.^23^ Neurons accumulate lipofuscin granules, have axonal loss, demyelination, and synapse number reduction and attenuated growth factor response.^24,25^ These changes result in age-related declines in nerve conduction velocity, muscle strength, sensory discrimination, and autonomic responses.^12,24^ As a result, nerve regeneration and reinnervation following peripheral nerve injury is significantly delayed and less effective with aging.^26^

## MECHANISMS OF AGING

**Fig. 4.**
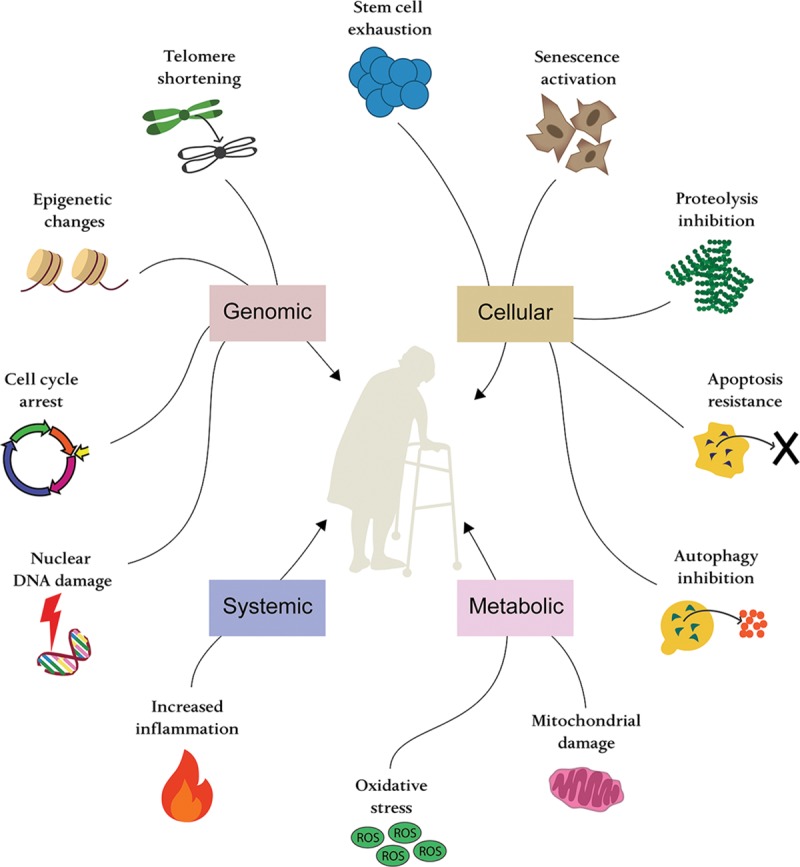
Mechanisms of aging. Aging occurs due to multiple intertwined mechanisms; dysregulation in proteostasis with subsequent accumulation of dysfunctional proteins, dysfunction of mitochondria and accumulation of reactive oxygen species, DNA and nuclear lamina damage, shortening of the telomeres, as well as dysregulation of intercellular signaling. All these mechanisms are believed to result in activation of cell senescence, inhibition of autophagy and apoptosis, and exhaustion of stem cells.

The understanding of molecular and cellular bases of aging has grown exponentially. Hallmarks of aging include genomic instability, telomere attrition, epigenetic alterations, loss of proteostasis, deregulated nutrient sensing, mitochondrial dysfunction, cellular senescence, stem cell exhaustion, and altered intercellular communication (Fig. 4).^27^ These areas are now active therapeutic targets to reverse age-related decline and associated pathology. Many of these therapies are in clinical trials and have relevance to the elderly undergoing surgery.

Age-related accumulation of unrepaired DNA damage leads to altered and dysfunctional gene expression and senescence, which drives cellular and tissue aging.^16,28–30^ Enhancing DNA repair is possible utilizing nicotinamide adenine dinucleotide–boosting molecules, which promotes health and extended lifespan in old and diseased animal models.^31,32^ These nicotinamide adenine dinucleotide–related molecules include sirtuins whose increased activity has been shown to promote longevity and healthy aging and are being tested in human clinical trials.^33^

Cellular senescence drives aging via accumulation of permanently growth-arrested senescent cells throughout multiple tissues. Senescent cells are apoptotic resistant, have altered gene expression, and produce aberrant inflammatory cytokines.^34^ Removing senescent cells in preclinical studies improved frailty, cardiac dysfunction, vascular dysfunction, diabetes, osteoporosis, vertebral disk degeneration, pulmonary fibrosis, and radiation-induced damage.^35^ Senolytic compounds are the focus on new biotechnology companies and an ongoing active area of investigation in human clinical trials.^35^

Stem cell exhaustion limits the aging body’s ability to regenerate.^36^ Intrinsic changes in adult stem cells incorporates almost all of the hallmarks of aging listed above. However, there are also extrinsic factors that are in play as systemic circulating factors have been shown to improve adult stem rejuvenation.^37^ Regardless, there is evidence that stem cell rejuvenation may be able to reverse the aging phenotype at the organismal level.^38^

## EFFECT OF AGING ON PLASTIC SURGERY OUTCOMES

Over one-third of U.S. surgical procedures are with patients >65 years, which will increase over time.^39^ Advanced age is consistently an independent risk factor for postoperative complications in general abdominal, cardiothoracic, vascular, and orthopedic surgery.^40,41^ Furthermore, frailty, an age-related cumulative decline in multiple physiological systems, has been shown to improve predictions of mortality and morbidity versus chronological age alone.^42–46^ Frailty scores are utilized in cardiac, oncologic, acute care, thoracic, vascular, burn, and orthopedic surgery to predict postoperative outcomes.^47–53^ In contrast, the overall effect of age and frailty of plastic surgery patients has yet to be fully characterized. There are few comprehensive studies that examine quality of life, recovery, and rehabilitation in aged plastic surgery patients.

### General Wound Healing and Cutaneous Repair

Alterations in wound healing pathways are well-documented with aging.^54,55^ Intrinsic wound healing in healthy elderly is delayed but not completely defective in optimal conditions. Regardless, age-related skin and wound healing changes and comorbidities predispose the elderly to nonhealing chronic wounds.^56^ About 10% of elderly patients in an acute care setting will develop a pressure ulcer during hospitalization.^57,58^ Margolis et al.^57^ calculated the pressure ulcer probability and compared with those 65–70 years, those >80 years were 4–20 times more likely to develop a pressure ulcer.

Although aging adversely affects wound healing timeframes, it may accelerate maturation and improve scar quality under optimal conditions.^54,59^ Bond et al.^60^ found that patients >55 years had accelerated maturation and improved incisional scars compared with younger patients. This may involve altered inflammation, an accelerated maturation phase,^54,60^ and age-related increased fibrillin and elastin during acute wound healing.^59^

**Fig. 5.**
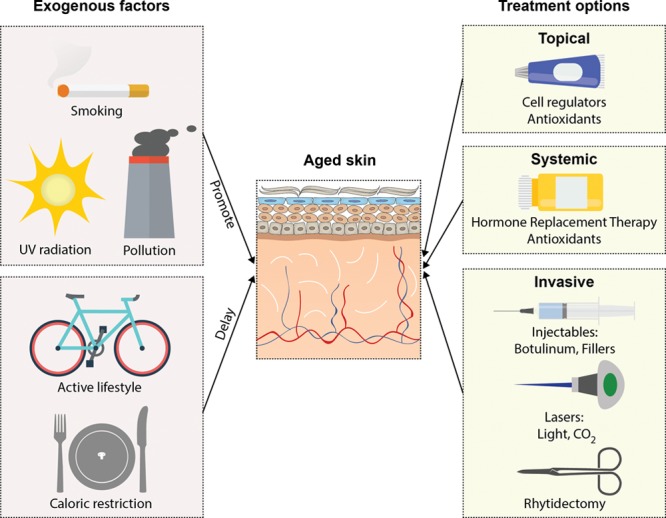
Skin aging treatment. In the preventative stage, there are numerous exogenous factors that are known to either promote or delay aging. Smoking, excessive sunlight exposure, stress, and pollution are all factors known to expedite aging. On the other hand, maintaining an active lifestyle, a low-calorie diet, as well as overall good health could help delay the aging process. Active treatment of aging skin is a multimodal approach, that can comprise medications, either topical or systemic, and invasive treatments that can be both nonsurgical and surgical.

Aged skin maintains regenerative capabilities as evidenced by skin rejuvenation therapies used on photoaged skin including laser resurfacing, microneedling, peels, and retinoic acid.^61,62^ Dermatologist and the cosmeceutical industry have long focused on nonsurgical skin rejuvenation,^63^ which can be used with surgical rejuvenation procedures (Fig. 5).^64,65^

### Wound Dehiscence

Age is an independent risk factor for surgical wound dehiscence in cardiothoracic, general abdominal, and orthopedic surgery.^66–69^ There are limited studies investigating surgical incision stability in plastic surgery. Karamanos et al.^70^ used NSQIP data to examine wound dehiscence from plastic surgery patients over five years. Their 0.75% dehiscence rate was no different in individuals >70 years and those younger. The authors, however, commented that high-risk elderly patients may have been counseled out of surgery and therefore not included.

### Breast Reconstruction

Multiple studies have examined age effects on breast reconstruction outcomes. A multicenter prospective study by Santosa et al.^71^ found that age >60 years did not significantly affect overall complication rates. However, this study found older women received significantly more delayed and unilateral reconstructions than younger patients. Butz et al.^72^ found that advanced age predicted 30-day complication after autologous but not implant-based reconstruction. A multicenter analysis of autologous reconstruction by Song et al.^73^ found that women >65 years only made 3% of total patients. Older women had similar patient satisfaction to younger patients and overall complications were similar. However, older women had statistically higher seroma and infection rates. Chang et al.^74^ found that >70 years was not associated with increased complications in microvascular breast reconstruction; however, the elderly cohort was only 2% of their study population and received mainly TRAM flaps (78%) and rarely DIEP flaps (5%). The complication rate of 43% in those >70 was increased compared with 28% in <70, however, not statistically significant. Selber et al.^75^ did not find increased complications in patients >65 years (only 5% of study population) undergoing free flap reconstruction in the immediate perioperative period.

There are mixed reports examining the effect of age on well-being, hospitalization, and morbidity after breast reconstruction. Johnson et al.^76^ found that women >65 years had similar self-reported well-being scores to those younger after reconstruction. Again, they found that older women were more likely to undergo unilateral and implant-based reconstruction than younger women. Girotto et al.^77^ found that patients >65 years scored worse than younger patients in postoperative physical function. Knackstedt et al.^78^ found that direct-to-implant in >65 years had similar complication rates with 2-stage reconstruction but significantly fewer readmissions, hospital stays, and postoperative visits. Abdominal donor-site morbidity after free flap reconstruction was found to be similar,^79^ which is in contrast to prior report stating decreased physical function in older women.^77^

### Trunk Reconstruction

A few studies have examined age in trunk reconstruction. Calotta et al.^80^ examined reconstruction of the posterior trunk after oncologic spine surgery and found no significant difference in complications between patients above or below 65 years. Giordano et al.^81^ found that abdominal wall reconstruction with acellular dermal matrix in >65 years has similar recurrence rates with <65, however, with more bulge/laxity rates.

### Craniofacial Trauma

Age-related craniofacial trauma is frequent in the elderly and often related to falls. Atisha et al.^1^ found that elderly patients >64 years generally required significantly less operative intervention and also had fewer surgical complications. However, Mundinger et al.^82^ found that patients >60 years had doubled length of hospitalization, were more likely to die, and be discharged to home with services.

### Hand Procedures

The impact of age on outcomes of hand/extremity procedures are challenging to define. Elderly upper extremity fractures are frequent and treatment algorithms are often different compared with younger patients. One example is closed treatment of distal radius fractures, which have remained the predominant treatment method in elderly patients.^83^ A systematic review by Diaz-Garcia et al.^84^ demonstrated that cast immobilization in elderly unstable distal radius fractures results in similar functional outcomes as surgically treated patients. However, a reported significant increase in distal radius fracture displacement rates associated with increasing patient age is likely related to impaired bony healing.^85^ With regard to other elective nontraumatic hand procedures, many of arthritis-related joint arthroplasties in the elderly population are due to increased incidence of osteoarthritis with age. Direct outcome comparisons between elderly and young patients are, thus, difficult to obtain.

Aging has detrimental effect on tendon repairs requiring more frequent reoperation. Dy et al.^86^ found that older patients were more likely to require reoperation after flexor tendon repair. Similarly Rigo and Røkkum^87^ found older age to be predictive of need for reoperation after tendon repairs.

Within limb and digital replantation, there are varying reports that comment on the impact of age on surgical outcomes. Barzin et al.^88^ found no differences in perioperative complications or mortality in patients >65 years undergoing digit replantation. However, patients >65 years required a higher rate of blood transfusion and discharge to rehabilitation facilities. A recent study by Kwon et al.^89^ found that digit replantation in patients >70 years was an important factor predicting replantation failure. Older patients had worse functional recovery than younger patients undergoing digital replantation.

### Peripheral Nerve Reconstruction

Age has significant detrimental impact on peripheral nerve repair outcomes. A meta-analysis of motor and sensory recovery after microsurgical repair of median and ulnar nerve injuries by Ruijs et al.^90^ demonstrated age as the main prognostic factor for recovery. Sensory recovery after digital replantation is also negatively impacted by increasing age. A systematic review by Glickman and Mackinnon^91^ found that younger patients had improved sensory recovery compared with older. This has been consistently demonstrated in the literature confirming the age-related decline in sensory recovery.^92–96^

A review on the efficacy of carpal tunnel release (CTR) found that the elderly had less predictable symptomatic and functional improvement following open CTR when compared with younger patients.^97^ CTR was found unlikely to result in the total elimination of symptoms when performed in elderly patients with advanced disease.^98^

As age increases, the axonal load of the facial nerve significantly decreases impacting facial reanimation procedures.^99^ Terzis and Konofaos^100^ found worse results in older patients undergoing facial reanimation. Her group has consistently found that there are significant age differences between the majority of patients with good to excellent results and cases that showed unsatisfactory results.^100,101^

### Microsurgical Reconstruction

The effect of age on microsurgical outcomes is controversial. Üstün et al.^102^ performed a systematic review and meta-analysis of free flaps in elderly patients. They found no difference in elderly versus young flap success rates or surgical complications, however, did find significantly more medical complications and mortality in elderly patients. Offodile et al.^103^ found age to be significant predictor of prolonged postoperative stay following free tissue transfer. Jubbal et al.^104^ analyzed 5,951 cases of free tissue transfer and found increased surgical and medical complication rates with increasing age. However, when controlling for comorbidities associated with age, age itself was not significantly associated with complications. Age was significantly associated with increased mortality. As such, the authors recommended assessment of “physiological” age instead of chronological age in assessing patients for free tissue reconstruction.

Lower extremity reconstruction in the elderly is more complex due to peripheral artery disease and diabetes. Xiong et al.^105^ found no difference in complication rates in patients >65; however, these patients required a higher rate of ICU admission. Surgical complications in retrospective studies are as high as 50% in free flaps and 40% in pedicled flaps with mean stays of 33 days and of 46 days, respectively.^106,107^

Age is consistently associated with complications in microvascular reconstruction of the head and neck in which patients often carry multiple medical comorbidities.^108–110^ Bhama et al.^111^ found older patients had significantly increased ICU stays and higher mortality rates. Blackwell et al.^112^ found that increased rate of complications and healthcare cost was nearly double in patients >80 years. Goh et al.^113^ did not find significant flap complications in patients >65 years, however, did find statistically significant increases in medical complications after free flap head and neck reconstruction. Chen et al.^114^ performed free flap reconstruction on patients >85 years and found a 37% surgical complication rate, noting high surgical and medical complications.

A number of studies have not found significantly increased free flap complications in elderly patients. Serletti et al.^115^ examined 100 patients >65 years retrospectively and found that chronological age did not predict flap complications. However, higher ASA scores and length of operative time were significant predictors of postoperative medical and surgical morbidity. Grammatica et al.^116^ performed a literature review of head and neck free flap reconstruction and found no difference in flap success, surgical complications, or mortality rate in elderly patients based on chronological age. They did acknowledge increased medical complications and proposed a comorbidity score to guide surgical planning. A systematic review of microsurgical scalp reconstruction by Sosin et al.^117^ also did find that chronological age did not increase mortality or catastrophic flap complications.

## CONCLUSIONS

Plastic surgery alters the aged appearance and reconstructs age-related wounds following cancer resection or trauma. In our review, we did not find a unifying consensus that defines the impact of age or frailty on plastic surgery outcomes. Instead, we hypothesize that these mixed reports are likely a product of both exclusion of aged and frail patients from surgery as well as outcomes that report “no issues” with aged patients in terms of reconstruction or flap survival. A more detailed examination of the postoperative morbidity, recovery course, rehabilitation progress, and patient-reported outcomes is needed.

Collaboration with our basic science and gerontology colleagues may help to incorporate new aging therapies and methods to improve on preoperative screening and conditioning as well as postoperative rehabilitation. Biomarkers for aging and frailty are currently being studied and validated.^118–121^ In addition, a comprehensive frailty scoring system will help identify areas to maximize success and recovery after major plastic surgery procedures. Prehabilitation before major surgery has shown to improve return to functional baseline, quality of life, decrease postoperative complications, and length of stay in randomized blinded controlled trials.^122–124^

Finally, as we continue to offer plastic and reconstructive surgery to our growing elderly population, a greater understanding of mechanisms and outcomes related to aging can promote desired outcomes by declining interventions on patients expected to not do well postoperatively and intervening on patients with elevated chronological age but not frailty.

## Supplementary Material
